# An aerobiological perspective of dust in cage-housed and floor-housed poultry operations

**DOI:** 10.1186/1745-6673-4-13

**Published:** 2009-06-10

**Authors:** Natasha Just, Caroline Duchaine, Baljit Singh

**Affiliations:** 1 Department of Veterinary Biomedical Sciences, Western College of Veterinary Medicine, University of Saskatchewan, Saskatoon, Canada; 2 Centre de Recherche de l'Institut Universitaire de Cardiologie et de Pneumologie de Québec, 2725 Chemin Sainte-Foy, Québec, Québec, Canada

## Abstract

The Canadian poultry production industry contributes nearly $10 billion to the Canadian economy and employs nearly 50,000 workers. However, modern poultry facilities are highly contaminated with airborne dust. Although there are many bioaerosols in the poultry barn environment, endotoxin is typically attributed with the negative respiratory symptoms observed in workers. These adverse respiratory symptoms have a higher prevalence in poultry workers compared to workers from other animal confinement buildings. Workers in cage-housed operations compared to floor-housed facilities report a higher prevalence of some respiratory symptoms. We review the current state of knowledge on airborne dust in poultry barns and respiratory dysfunction in poultry workers while highlighting the areas that need further investigation. Our review focuses on the aerobiological pathway of poultry dust including the source and aerosolization of dust and worker exposure and response. Further understanding of the source and aerosolization of dust in poultry operations will aid in the development of management practices to reduce worker exposure and response.

## Review

In 2007, chicken held the largest share (33.2%) of consumed meat by Canadians. The industry is nation-wide, with facilities in every province. The Canadian poultry industry contributes up to $9.5 billion to the Canadian economy, creates a total of 49,700 jobs and generates $1.78 billion in wages and personal income [1]. These facts highlight the importance of poultry production in Canada. Modern methods of poultry facility management require that workers spend a large proportion of the day in an atmosphere containing comparatively high levels of dust, gases and odors [2,3]. Poultry farmers have a high exposure to microbial products and components such as endotoxin, β-glucan and peptidoglycan [3-5]. Studies of different industries showed the highest prevalence of work-related lower and upper respiratory symptoms and lower baseline lung function in poultry workers [5,6]. Workers typically complain of chronic cough that may be accompanied by phlegm, eye irritation, dyspnea, fatigue, headache, nasal congestion, fever, throat irritation, chest tightness and wheezing [6-8]. Clinical diseases observed in poultry workers include allergic and non-allergic rhinitis, organic dust toxic syndrome (ODTS), chronic bronchitis, hypersensitivity pneumonitis (Farmer's Lung), toxin fever and occupational asthma or asthma-like syndrome [3,5,9,10].

Cage-housed and floor-housed operations are two common types of poultry housing facilities. In cage-housed operations birds are housed in cages for egg production and in floor-housed operations birds are housed on the floor for meat production. There are a number of differences in the two types of poultry operations including time spent by the workers in direct contact with birds, predominance of female poultry in cage-housed facilities, age of birds, length of time birds spend in housing and housing management practices. Previous data show that personal total dust exposures are significantly higher in floor-housed versus cage-housed operations [2,6]. However, a trend towards higher endotoxin concentration (EU/mg) in cage barns was observed [6]. Significant differences in symptoms are observed between cage-housed and floor-housed workers. Current and chronic phlegm occurred more frequently in workers from cage-housed facilities. Endotoxin concentration (EU/mg) is shown to be a significant predictor of chronic phlegm [6]. Therefore, type of housing may influence levels of environmental contaminants in the dust.

**Figure 1:**
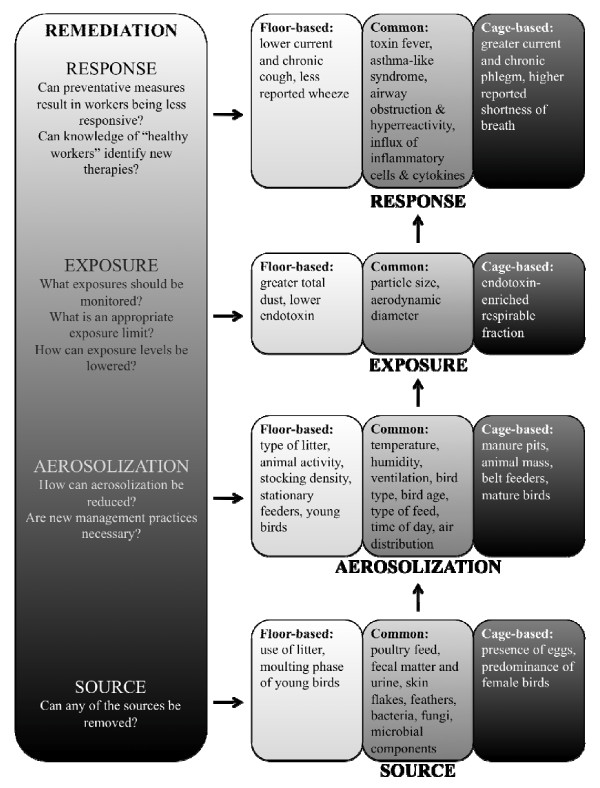
**Aerobiological pathway of dust in poultry facilities**. Common factors influencing each stage of the pathway are indicated in the grey boxes, specific cage-housed factors are highlighted in black boxes and floor-housed factors are outlined in white boxes. Remediation opportunities for each stage of the pathway are indicated at the left.

A better understanding of the poultry house environment is needed to improve the respiratory health of poultry workers. The aerobiological pathway that results in dust production includes the source, aerosolization and dispersal, exposure, response and remediation (Figure 1). Elucidation of this pathway will help identify means of prevention and/or treatment of the respiratory symptoms observed in poultry workers. Examination of the two types of poultry operations separately may reveal different means of improving respiratory health in the two types of workers.

## Sources

Dust is a complex mixture of particles of organic and inorganic origin and different gases absorbed in aerosol droplets. The sources of dust from a poultry facility include dried fecal matter and urine, skin flakes, ammonia, carbon dioxide, pollens, feed and litter particles, feathers (which produce allergen dandruff), grain mites, fungi, spores, bacteria, viruses and their constituents, peptidoglycan, β-glucan, mycotoxin and endotoxin [3,6,11-13]. Endotoxin is the most frequently reported environmental contaminant in poultry dust. Endotoxin is the family of lipopolysaccharide (LPS) fragments that coat the outer membrane of Gram-negative bacteria [14]. LPS is composed of three structural elements: a core oligosaccharide, an O-specific chain made up of repeating sequences of polysaccharides and a lipid A component, which is responsible for the toxic effects of LPS exposure [15]. Common occupational sources of exposure include livestock, grain dust, and textiles, but significant concentrations also occur in the household from pets, carpeting and indoor ventilation systems. Endotoxin has also been found in tobacco smoke and particulate matter in air pollution [14]. In poultry operations, endotoxin originates from bacteria that can be found in fecal matter, urine, litter, grain and other vegetable matter in poultry feed [3,16,17]. Endotoxin can be measured by the Limulus amoebocyte lysate-based (LAL) bioassay, which measures biological activity of endotoxin, or by mass spectrometry, which can quantify endotoxin biochemically through detection of LPS-characteristic 3-hydroxy fatty acids [18].

Airborne and settled poultry dusts have similar chemical compositions. One study showed approximately 900 g/kg dry matter, 95 g/kg ash, 150 g/kg nitrogen, 6.5 g/kg phosphorous, 30 g/kg potassium, 4 g/kg chlorine and 3 g/kg sodium. Down feathers and crystalline dust are the major physical components of dust. Crystalline dust originates from urine [12]. The solid components of dust act as a transport vector for noxious gases and biological contaminants, allowing these to be inhaled into the lungs [19].

Organic dust components can be further divided into non-viable and viable particulate matter, or bioaerosols [11]. Microorganisms represent less than 1% of airborne particles but are often associated with the negative health effects associated with the poultry industry [19]. The aerobic bacteria common in poultry facilities include: *Bacillus *sp., *Micrococcus *sp., *Proteus *sp., *Pseudomonas *sp., *Staphylococcus *sp. and *E. coli *and common anaerobic bacteria are *Clostridia *sp. [20]. Experimental poultry houses showed that 80% of airborne bacteria were Gram-positive aerobes and only 7–17% were Gram-negative rods when litter was present. However, approximately 40% of the Gram-negative bacteria can be trapped in the respirable fraction of dust using an Andersen sampler. Coliform bacteria have low viability in the air and so are more common in litter [3]. Airborne fungi present in poultry facilities include *Cladosporium *sp., *Aspergillus *sp., *Penicillium *sp. and less commonly, *Alternaria *sp., *Fusarium *sp., *Geotrichum *sp. and *Streptomyces *sp. [20,21].

Types and levels of fungi and bacteria depend on management processes that control relative humidity, temperature, type and age of the litter and the source, which may already be present in the building [3]. In floor-housed operations it has been shown that levels of airborne dust, endotoxin and bacteria increase throughout the growth cycle of the chickens [11]. This increase parallels the increase of biomass (number of birds × bird weight) during the growth cycle and corresponding higher levels of skin debris and feathers.

Typically, the incidence of microorganisms is reported as CFU/m^3 ^air. Reported incidences in poultry environments include 3.4 ± 1.4 × 10^5 ^CFU/m^3 ^for culturable bacteria and 2.8 ± 2.1 × 10^4 ^CFU/m^3 ^for culturable fungal spores [21]. However, recent results show that culture-dependent techniques underestimate total bacteria or total fungi measured by culture-independent approaches such as epifluorescence and quantitative PCR [22]. The measure of total fungi in poultry operations is 2.0 × 10^7^/m^3 ^and measures of total bacteria range from 5.3 × 10^8^/m^3 ^to 4.7 × 10^9^/m^3 ^[5,11].

Antimicrobials are used for growth promotion, disease prevention and treatment of illnesses in the poultry industry. Some of these antimicrobials are similar or identical in chemical structure to antimicrobials used to treat human infections [23]. The approval for use of antimicrobials is in question for various reasons. Antimicrobial resistance genes have been isolated from poultry bacteria such as *Salmonella *sp., *Campylobacter *sp. and *E. coli *[24]. Some of these bacteria are human pathogens and antimicrobial-resistant bacteria can be transferred to humans, which is a health concern. For example, fluoroquinolone-resistant *Campylobacter *in poultry operations is transferred to humans and causes fluoroquinolone-resistant *Campylobacter *infections [23].

Characterization of dust sources is important in order to identify those that may, or may not, be removed (Figure 1). For example, endotoxin originates from bacteria found in fecal matter, urine, litter and feed particles. Although the presence of feces, urine, litter and feed are all intrinsic to poultry production, the types of feed and litter may alter the types and levels of bacteria, providing a potential means for lowering sources of endotoxin.

## Aerosolization and dispersal

The contaminants described in the preceding section can be readily aerosolized and dispersed throughout the poultry barn environment. Aerial dust concentrations are affected by the rate of aerosolization, settling velocities and resuspension rates of airborne particles [19]. Therefore, aerosol concentrations in animal confinement buildings are dependent on animal activity, air temperature, relative humidity, ventilation rate, animal stocking density, animal mass, type of litter, type of bird, bird age, type of feed, feeding method, time of day, air distribution, relative locations of dust sources and presence or absence of air cleaning technologies [3,12].

Microorganisms exist suspended in the air as well as attached to dust particles. The survival time for bacteria is affected by many factors: mechanism of dispersal into the air, deposition on host surfaces, host susceptibility, humidity, temperature, bacterial repair processes and the open-air factor, which can kill microorganisms. Therefore, management practices can directly affect the levels of bacteria. For example, increasing the stocking density and temperature of poultry facilities leads to an increase in the concentrations of airborne organisms [3].

Circulating fans move the air throughout the barn while ventilation fans move air across the barn. Contaminated indoor air is expelled from animal facilities by exhaust fans. *E. coli *and *Salmonella *were isolated up to 12 m from poultry facilities. At 3 m from poultry building exhaust fans, dust concentrations can be relatively high (32–75 mg/m^3^) but fall below 2 mg/m^3 ^by 12 m from ventilation fans [13]. Vents located along the walls and in the roof allow for outdoor air intake. Outdoor air contains endotoxin due to aerosolization of Gram-negative bacteria from leaves. Outdoor endotoxin can contribute to indoor levels due to the high outdoor air intake of animal facilities [13].

An increased ventilation rate will not necessarily reduce overall dust concentrations since the dust production rate increases with increased ventilation. Dust levels depend on relative humidity. Less ventilated buildings have high relative humidity and lower dust aerosolization than highly ventilated buildings. However, in buildings with natural ventilation or extremely high ventilation rates, dust levels drop [19]. Adjustment of relative humidity to 75% will have an effect on inhalable dust (the fraction that is below 20 μm), but not on respirable dust (the fraction below 5 μm) [12]. However, litter moisture increases during periods of high humidity and ammonia levels increase with litter moisture [12].

Mechanical disturbance by animal movement is the prime method of aerosolization in poultry facilities. If light programs are used, dust concentrations are much lower at night than during the day due to less animal movement [12]. Aerosolization of organic dust particles and endotoxin varies between the two poultry barn types. There is less ground disturbance in facilities where birds are not housed on the floor and movement is restricted.

The type of flooring and litter used in the facility alters aerosolization of dust particles [13]. Generally, dust concentrations are lowest in cage-housed facilities that use manure collection systems and are highest in floor-housed operations that use litter as bedding material. At 32°C, the rate of dust production in floor-housed operations decreases to that of cage-housed facilities. This is attributed to an increase in humidity, which decreases the generation rate of dust from floor litter and causes airborne dust to settle more rapidly [3]. There is a predominance of female birds as well as different bird types in cage-housed versus floor-housed operations. In floor-housed operations it is expected that aerosolization of dust increases throughout the chicken growth cycle [11]. Young birds undergo molting, which contributes to large particle production during this time of development. Birds enter floor-housed operations at approximately one week of age and are removed by approximately 40 days of age. However, birds enter cage-housed facilities at approximately twenty weeks of age and continue laying eggs until approximately 70 weeks of age. These differences coincide with observations of greater dust concentrations in floor-housed poultry facilities.

Many management practices have been identified that influence aerosolization and dispersal of dust (Figure 1). Using the optimal practices for lowering aerosolization is a potential means for lowering dust exposure in poultry operations.

## Exposures

Aerosolization of dust particles into the breathing zone of workers results in exposure to bioaerosols. Dust particles vary in size and shape in animal confinement buildings [19]. Differentiation between particle size fractions is important in health studies in order to quantify penetration of dust within the respiratory system. Particles of similar size but different shape and density behave differently in air. Therefore, 'aerodynamic diameter' is used to describe the size of particles that behave similarly to spheres of unit density. Particles with high density tend to have a high settling velocity, whereas less dense particles will remain airborne longer.

Particles of all sizes may be deposited in the nose and pharyngeal region. However, only particles with an aerodynamic diameter of less than 15 μm can enter the tracheobronchial tree and only particles with an aerodynamic diameter of less than 7 μm can enter the alveoli [3]. Approximately 50% of particles less than 5 μm aerodynamic diameter entering the respiratory system will reach the alveoli. Therefore, the fraction of dust including particles less than 5 μm aerodynamic diameter is the respirable fraction [3]. The particle size range with the largest percentage of deposition in the lungs is 1–2 μm in aerodynamic diameter. Respirable dust accounts for ~18% of total dust mass [3]. Particles smaller than 0.5 μm in mean aerodynamic diameter are respirable, but it is more likely that they are exhaled and not deposited in the lungs. Therefore, interest lies in controlling "modified" respirable dust, 0.5–5 μm, and "modified" inhalable dust, >5 μm in mean aerodynamic diameter [25].

Dust concentrations in poultry facilities can range from 0.02 to 81.33 mg/m^3 ^for inhalable dust and 0.01 to 6.5 mg/m^3 ^for respirable dust. Cage-housed facilities show the lowest dust concentrations, <2 mg/m^3^, while dust concentrations in floor-housed operations are typically four to five times higher [12]. Endotoxin levels are also typically higher for cage-housed versus floor-housed operations [6]. Endotoxin concentration of respirable dust, 20 to 40 ng/mg, is considerably higher than endotoxin concentration of total dust, 6 to 16 ng/mg, suggesting that endotoxin is enriched in smaller particles [26]. It is hypothesized that fine particle concentrations differ between the two types of poultry facilities. The lower total dust in cage barns could be a result of more fine particles with lower mass but larger surface area, carrying more endotoxin that is able to remain aerosolized longer and penetrate deeper in the lung [6]. Interactions between endotoxin and the lung result in negative respiratory and immune responses.

As mentioned above, dust is a complex mixture of both viable and non-viable sources, including endotoxin, bacteria and fungi. Therefore, monitoring of several types of exposures is necessary. Characterizing typical exposure levels to each of these contaminants is required to help set exposure limits and find means of lowering exposures, for potential remediation (Figure 1).

## Worker response

The following lung function measurements are used during the assessment of respiratory health: forced expiratory volume in 1 second (FEV_1_), forced vital capacity (FVC), and forced expiratory flow rate between 25 and 75% of FVC (FEF_25–75_). Decreases in FEV_1_, FVC and FEF_25–75 _are normally indicative of obstructive ventilation caused by narrowing of the airways. Restrictive disorders are caused by changes in compliance of lung tissues or the chest wall [3]. A relationship has been shown between respiratory insult to known endotoxin concentrations and change in FEV_1_. Cross-shift declines in FEV_1_, FVC and FEF_25–75 _have been identified and correlate to endotoxin exposure in the workplace. Cross-shift changes have also been shown to predict longitudinal changes in lung function [27].

Exposure to endotoxin causes episodic febrile reactions. Toxin fever generally occurs in the afternoon or evening of a working day. Symptoms of toxin fever include: headache, nausea, coughing, nasal irritation, chest tightness and phlegm. The minimum level of endotoxin required to produce a fever reaction in humans is 0.5 μg/m^3 ^following a four-hour exposure period [3]. Endotoxins derived from different species of Gram-negative bacteria differ in their toxicity. Therefore, the minimum level required to produce fever is species-dependent.

Inhalation of endotoxin can cause many physiological airway responses including airflow obstruction, enhanced airway hyperreactivity and a reduction in alveolar diffusion capacity. Bronchoalveolar lavage (BAL) fluid following endotoxin instillation shows increased numbers of macrophages and neutrophils along with increased concentrations of interleukin-6 (IL-6), IL-8, IL-1β, and tumor necrosis factor (TNF-α) [28].

Exposure to the confinement barn environment can cause acute and chronic respiratory symptoms in workers similar to those observed following endotoxin inhalation. Workers typically complain of chronic cough that may be accompanied by phlegm, eye irritation, dyspnea, fatigue, headache, nasal congestion, fever, throat irritation, chest tightness, shortness of breath with exertion and wheezing [6-8]. Clinical diseases observed in poultry workers include allergic and non-allergic rhinitis, organic dust toxic syndrome (ODTS), chronic bronchitis, hypersensitivity pneumonitis (Farmer's Lung), toxin fever and occupational asthma or asthma-like syndrome [3,5,9,10]. Significant differences in symptoms are observed between cage-housed and floor-housed workers. Current and chronic phlegm occurred more frequently in workers from cage barns. Endotoxin concentration (EU/mg) is shown to be a significant predictor of chronic phlegm [6]. However, the symptoms generated by poultry dust are thought to be non-specific and caused by a variety of agents, which makes it difficult to find a dose-response relationship or set exposure limits [3].

The literature contains more response data to swine barn environment exposure than poultry barn environment exposure. Naïve subjects exposed to the swine barn environment have been shown to develop symptoms such as cough, dyspnea, nasal stuffiness, headache, fever and chills, malaise, nausea and eye irritation after several hours of exposure. Following acute exposure, these naïve subjects also show airway hyperresponsiveness characterized by a decline in peak expiratory flow rates and FEV_1_[27]. Continued exposure for only a short period of time (weeks) can increase this bronchial hyperresponsiveness and lead to occupational asthma. The "healthy worker effect" is the phenomenon where individuals seriously affected by occupational asthma-like symptoms leave the industry following only a short exposure period [29]. Further detailed knowledge on the lung function of "healthy workers" is required.

Adaptation occurs when repeated exposures result in a reduced injury response compared to a single exposure alone. There is evidence to support an adaptive response to endotoxin exposure in animal confinement workers. A lower number of inflammatory cells is recovered from the lower respiratory tract of workers compared to naïve subjects and a smaller decline in lung function and reduced bronchial responsiveness to methacholine is observed in workers versus naïve controls [27]. Genetic factors, such as Toll-like receptor (TLR) mutations, also play a role in endotoxin tolerance.

Most LPS moieties activate cells through binding TLR4. However, LPS from some bacterial species, such as *P. gingivalis*, activate cells through TLR2 binding. A polymorphism of TLR4 (Asp299Gly) is observed in approximately 10% of individuals in the general population and has been associated with a blunted response to LPS in vitro and with a diminished airway response to inhaled LPS [14]. This missense mutation alters the extracellular domain of the TLR4 receptor. An additional polymorphism (Thr399Ile) co-segregates with the Asp299Gly substitution [30]. Co-segregating missense mutations are also associated with a blunted response to inhaled LPS in humans. These results indicate the importance of other genetic and/or environmental factors in determining response to inhaled endotoxin and a need for further studies to understand the mechanisms.

It is hypothesized that "healthy workers" have a diminished response to dust contaminants, including endotoxin, through genetic factors. Further understanding of the genetics that result in hyporesponsiveness may lead to potential means of remediation, by treating or preventing the worker response in non-healthy workers (Figure 1).

## Remediation

The overwhelming evidence of the negative respiratory symptoms and immunological effects of poultry dust exposure suggests a need for remediation. However, many sources of dust, including some sources of endotoxin, are intrinsic to the poultry production industry and therefore, remediation is difficult (Figure 1). Keeping poultry facilities clean has long been encouraged as a method to protect human respiratory health. Adopting management practices such as use of pelleted food, routine entry into buildings and use of lighting cycles can control dust and ammonia levels. However, some practices may lower one contaminant while increasing another. For example, dry litter reduces ammonia production but is aerosolized more easily by animal activity. Also, application of water mists can reduce dust production by increasing the settling velocity of airborne particles but increases relative humidity, which facilitates ammonia production [3]. Both the use of well-fitted N-95 respirators by workers and spraying water or oil mixtures to reduce dust are shown to be effective at reducing dust exposure in animal confinement buildings [12,19,25,31,32]. Although spraying water is useful at reducing dust production, it increases relative humidity, which facilitates microbial growth [3].

Altering management practices may be a means of reducing aerosolization of barn contaminants, thus reducing worker exposure. Understanding the levels of worker exposures to bioaerosols may help introduce new management practices to reduce exposure, such as better personal protective equipment. Bettering understanding of the workers response may lead to new means of treatment (Figure 1). Examining the environmental differences between cage-housed and floor-housed poultry operations may provide insight into other means of remediation.

## Conclusion

Dust sources, including endotoxin, are present at high concentrations in poultry facilities. The aerobiological pathway of poultry dust is outlined in figure 1. Endotoxin can be recovered from air samples due to its association with dust particles. The production of poultry dust can vary due to factors including: animal activity, air temperature, relative humidity, ventilation rate, animal stocking density, type of litter, type of bird, bird age, type of feed, feeding method, time of day, air distribution, relative locations of dust sources and presence or absence of air cleaning technologies [3,12]. Also, particle size is a key factor in poultry dust production since rate of aerosolization, settling velocity and resuspension rate of airborne particles differ depending on particle size [19].

Dust production is typically higher in floor-housed versus cage-housed poultry facilities [6]. Management practices differ between the two types of poultry facilities. Animal activity is higher in floor-housed operations where birds move freely as opposed to being housed in cages. This higher level of activity contributes to greater particle aerosolization. Litter is a source of dust production and is used in floor-housed operations but not in cage-housed facilities. The predominance of female birds in cage-housed operations as well as different bird types contribute to differences in the air environment. Bird age is also a factor that differs between the two barn types and has an effect on bioaerosols. These differences coincide with observations of greater dust concentrations in floor-housed poultry facilities.

Interestingly, observations of higher total dust concentrations in floor-housed operations are not in agreement with the observations of greater respiratory dysfunction in cage-housed workers. Further investigation of dust concentrations at different size fractions suggests that cage-housed operations have higher concentrations of respirable dust than floor-housed facilities [6]. A Canadian study looking only at particles less than 5 μm in diameter showed the opposite results. Cage barns had higher particle levels than floor barns at 40 particles/mL and 7–27 particles/mL, respectively [6]. Particles of respirable size remain airborne longer than larger particles due to higher rate of aerosolization and lower settling velocity. These particles also penetrate deeper within the respiratory system. Therefore, the higher concentrations of smaller dust particles in cage-housed facilities may be responsible for the more negative health effects observed, even in the presence of lower total dust concentrations.

A better understanding of the barn air environment, including bioaerosols, is required to reduce aerosolization and dispersal, decrease worker exposure and prevent or treat respiratory symptoms. Further examination of the aerobiological pathway will help to find means of remediation. Since particle size is an important factor for aerosolization, further research into bioaerosol contamination at different particle size fractions is necessary. Viable microorganisms contributing to bioaerosol production have been identified. However, methods to identify the contributions of non-viable microbes are required. In swine facilities, some forms of remediation have been tested. These methods include the use of respirators by workers and spraying of canola oil to reduce dust exposure. Such methods need to be evaluated in the poultry industry. The economic importance of maintaining the poultry production industry is obvious. However, the respiratory dysfunction of poultry workers is a major health issue and requires detailed investigation.

## Abbreviations

BAL: bronchoalveolar lavage; CFU: colony forming unit; EU: endotoxin unit; FEF_25–75_: forced expiratory flow rate between 25 and 75% of FVC; FEV_1_: forced expiratory volume in 1 second; FVC: forced vital capacity; IL-1β: interleukin-1 beta; IL-6: interleukin-6; IL-8: interleukin-8; LAL: Limulus amoebocyte lysate; LPS: lipopolysaccharide; ODTS: organic dust toxic syndrome; PCR: polymerase chain reaction; sp.: species; TNF-α: tumor necrosis factor-alpha; TLR2: toll-like receptor 2; TLR4: toll-like receptor 4

## Competing interests

The authors declare that they have no competing interests.

## Authors' contributions

NJ participated in drafting the manuscript. CD and BS participated in revising the manuscript. All authors have read and approved the final manuscript.
